# Therapeutic Role of Retroperitoneal Lymphadenectomy in 170 Patients With Ovarian Clear Cell Cancer

**DOI:** 10.3389/fonc.2021.754149

**Published:** 2022-01-13

**Authors:** Wen Gao, Peipei Shi, Haiyan Sun, Meili Xi, Wenbin Tang, Sheng Yin, Jiarong Zhang

**Affiliations:** ^1^ Department of Gynecologic Oncology, The Cancer Hospital of the University of Chinese Academy of Sciences (Zhejiang Cancer Hospital), Institute of Basic Medicine and Cancer (IBMC), Chinese Academy of Sciences, Hangzhou, China; ^2^ Department of Obstetrics and Gynecology, Zhongshan Hospital, Fudan University, Shanghai, China

**Keywords:** ovarian clear cell cancer, retroperitoneal lymphadenectomy, cancer stage, progression free survival, overall survival

## Abstract

**Introduction:**

We evaluated the therapeutic role of retroperitoneal lymphadenectomy in patients with ovarian clear cell cancer (OCCC).

**Materials and Methods:**

We retrospectively reviewed 170 OCCC patients diagnosed at two hospitals in China between April 2010 and August 2020. Clinical data were abstracted, and patients were followed until February 2021. Patients were divided into retroperitoneal lymphadenectomy and no lymphadenectomy groups. The Kaplan–Meier method was used to compare progression-free (PFS) and overall survival (OS) between the two groups. Statistical differences were determined by the log-rank test. The COX proportional hazards regression model was applied to identify predictors of tumor recurrence.

**Results:**

The median age was 52 years; 90 (52.9%) and 80 (47.1%) patients were diagnosed as early and advanced stage, respectively. Clinically positive and negative nodes was found in 40 (23.5%) and 119 (70.0%) patients, respectively. Of all the 170 patients, 124 (72.9%) patients underwent retroperitoneal lymphadenectomy, while 46 (27.1%) did not. The estimated 2-year PFS and 5-year OS rates were 71.4% and 65.9% in the lymphadenectomy group, and 72.0% and 73.7% in no lymphadenectomy group (p = 0.566 and 0.669, respectively). There was also no difference in survival between the two groups when subgroup analysis was performed stratified by early and advanced stage, or in patients with clinically negative nodes. Multivariate analysis showed that retroperitoneal lymphadenectomy were not an independent predictor of tumor recurrence.

**Conclusion:**

Retroperitoneal lymphadenectomy provided no survival benefit in patients diagnosed with OCCC. A prospective clinical trial is needed to confirm the present results.

## Introduction

Epithelial ovarian cancer (EOC) is the most lethal of all gynecologic malignancies. In 2020, the estimated number of deaths was 13 940 in the USA, which ranks fifth in cancer deaths among women (1). Ovarian clear cell cancer (OCCC) is a lethal histological subtype with an incidence rate ranging from 5%–25% according to geographical area and race (2).

Although the distinct biological and clinical behavior of OCCC differs extensively from serous ovarian cancer, such as younger age and earlier International Federation of Gynecology and Obstetrics (FIGO) stage at diagnosis, greater chemoresistance, and higher rate of thromboembolic complications, the surgical treatment of these different EOC subtypes is similar (2, 3). According to the National Comprehensive Cancer Network (NCCN) clinical practice guidelines for ovarian cancer/Fallopian tube cancer/primary peritoneal cancer (Version 1. 2021, available at NCCN.org), standard surgical staging procedures, including systematic retroperitoneal lymphadenectomy (para-aortic and pelvic lymph nodes) should be performed in ovarian cancer patients with early FIGO stage (apparent FIGO stage IA–IIA). For patients with advanced ovarian cancer involving the pelvis and upper abdomen (FIGO stage ≥ IIB), optimal cytoreductive surgery, including resection of suspicious and/or enlarged nodes, should be performed, while this is not required for patients with clinically negative nodes.

Previous studies have shown inconsistent results regarding the prognostic impact of retroperitoneal lymphadenectomy for ovarian cancer in both early- and advanced-stage patients (4–8). Furthermore, different ovarian cancer subtypes have distinct biological and clinical behavior, which is especially true for OCCC; therefore, the subtypes should be studied separately. We conducted this retrospective study to estimate the prognostic impact of retroperitoneal lymphadenectomy in patients with OCCC.

## Methods and Materials

### Patients

This was a retrospective study conducted at Fudan University Zhongshan Hospital and Zhejiang Cancer Hospital between April 2010 and August 2020. Patients who were primarily treated and pathologically diagnosed with OCCC were identified, and their clinical data were collected.

Medical records were abstracted to obtain the patients’ age at diagnosis; preoperative value of serum carbohydrate antigen (CA)125 and CA199; preoperative imaging; FIGO stage; preoperative venous thromboembolism (VTE); type of surgery (laparotomy or laparoscopy); Fagotti score; ascites volume; intraoperative exploration; surgical procedures; pathology of dissected lymph nodes; adjuvant chemotherapy; number of chemotherapy cycles; residual disease after primary surgery; and PFS and OS.

Due to the retrospective nature of this study, there were no standards for performing retroperitoneal lymphadenectomy between different surgeons in the two centers. Normally, patients would receive retroperitoneal lymph node dissection when clinically positive nodes were identified according to preoperative imaging or intraoperative exploration. However, for patients with clinically negative nodes, whether to perform lymphadenectomy or not would be determined by the surgeons. Overall, we divided the patients into two groups: lymphadenectomy group and no lymphadenectomy group. Lymphadenectomy group included patients receiving systematic lymph node resection (systematic pelvic lymphadenectomy with or without para-aortic lymphadenectomy or biopsy) and partial lymph node dissection (few patients with enlarged para-aortic lymph node received para-aortic lymph node resection only). Patients did not undergo lymph node resection were included in no lymphadenectomy group. To analyze the role of lymphadenectomy, subgroup analysis was performed stratified by early-stage (FIGO stage IA–IIA) and advanced-stage (FIGO stage IIB–IVB), and also in patients with clinically negative nodes.

The study was approved by the medical ethics committees of both Fudan University Zhongshan Hospital (B2021-368) and Zhejiang Cancer Hospital (IRB-2021-244). PFS was defined as the time from primary surgery to the date of recurrence, and OS was calculated as the time from primary surgery to the date of death or the last follow-up. The last follow-up date was in February 2021.

### Statistical Analysis

The SPSS software package for windows (version 19.0; SPSS Inc., Armonk, NY, USA) was used for statistical analysis. The Chi-square or Mann-Whitney U tests were used to identify differences in the baseline level between lymphadenectomy and no lymphadenectomy group. The Kaplan–Meier method was used to compare survival between groups, and statistical differences were determined by the log-rank test. The COX proportional hazards regression model was applied to identify prognostic factors. A *p*-values of < 0.05 was considered statistically significant.

## Results

### Baseline and Clinical Characteristics

We enrolled 170 patients in this study, namely 43 patients from Fudan University Zhongshan Hospital and 127 patients from Zhejiang Cancer Hospital. Clinical characteristics of the 170 patients was shown in **Supplementary Table 1**. The median age at diagnosis was 52 years (range, 30–79 years). More than half of the patients (52.9%) were diagnosed with early-stage disease (FIGO stage IA–IIA). Clinically positive and negative nodes were found in 40 (23.5%) and 119 (70.0%) patients, respectively. In 119 patients with clinically negative nodes, 79 (66.4%) and 40 (33.6%) patients were included in lymphadenectomy and no lymphadenectomy group, respectively. In total, 124 (72.9%) patients underwent lymphadenectomy, while 36 (27.1%) did not. The patients’ baseline characteristics in the lymphadenectomy and no lymphadenectomy groups are shown in **Table 1**, and the baseline characteristics were well balanced except regarding residual disease. In the no lymphadenectomy group, patients tended to undergo suboptimal surgery.

**Table 1:** Clinical characteristics between lymphadenectomy and no lymphadenectomy group.

Characteristics	Lymphadenectomy group (n = 124)	No lymphadenectomy group (n = 46)	*P* value
Age at diagnosis			
≤50	59 (47.6%)	15 (32.6%)	0.085
>50	65 (52.4%)	31 (67.4%)	
Median preoperative CA125 (U/ml)	137.4	219.0	0.430
Median preoperative CA19-9 (U/ml)	25.2	24.5	0.212
FIGO Stage			
Early (IA-IIA)	67 (54.0%)	23 (50.0%)	
Advanced (IIB-IVB)	57 (46.0%)	23 (50.0%)	0.730
Lymph node status			
Clinically positive	37 (29.8%)	3 (6.5%)^a^	
Clinically negative	79 (63.7%)	40 (87.0%)	
NA	8 (6.5%)	3 (6.5%)	0.001
VTE			
Yes	8 (6.5%)	7 (15.2%)	
No	116 (93.5%)	39 (84.8%)	0.123
Fagotti score			
<8	114 (91.9%)	38 (82.6%)	
≥8	10 (8.1%)	8 (17.4%)	0.095
Ascites			
None	74 (59.7%)	28 (60.9%)	
Yes	47 (37.9%)	16 (34.8%)	
NA	3 (2.4%)	2 (4.3%)	0.857
Residual disease			
NGR	111 (89.5%)	34 (73.9%)	
RD >0	10 (8.1%)	10 (21.7%)	
NA	3 (2.4%)	2 (4.3%)	0.028
Chemotherapy			
Taxane + platinum	109 (87.9%)	39 (84.8%)	
Other platinum-based chemotherapy	4 (3.2%)	3 (6.5%)	
Others	2 (1.6%)	0	
None	7 (5.6%)	4 (8.7%)	
NA	2 (1.6%)	0	0.547
Chemotherapy cycles			
0-3	28 (22.6%)	13 (28.3%)	
≥4	94 (75.8%)	33 (71.7%)	
NA	2 (1.6%)	0	0.546

CA125, carbohydrate antigen 125; CA19-9, carbohydrate antigen 19-9; FIGO, International Federation of Gynecology and Obstetrics; VTE, venous thromboembolism; NGR, no gross residual disease; RD, residual disease; NA, not available.

a Three patients did not received retroperitoneal lymph node resection because of suboptimal debulking surgery in abdominal cavity (residual disease >1cm).

### Pathological Characteristics

Of the 124 patients undergoing retroperitoneal lymphadenectomy, 36 (29.0%) patients underwent pelvic lymph node resection, 5 patients (4.0%) underwent aortic lymph node resection, and 83 (66.9%) patients underwent both pelvic and aortic lymph node resection. Postoperative pathology of the dissected lymph nodes showed that 27 (21.8%) patients had positive lymph nodes, and 97 (78.2%) patients had negative lymph nodes. Forty-nine (39.5%) and 72 (58.1%) patients had < 20 and ≥ 20 lymph nodes resected, respectively (**Table 2**).

**Table 2:** Lymphadenectomy characteristics.

Characteristics	n = 124
Lymph node dissection	
Pelvic only	36 (29.0%)
Aortic only	5 (4.0%)
Pelvic and aortic	83 (66.9%)
Lymph node metastasis	
Positive	27 (21.8%)
Negative	97 (78.2%)
Number of lymph node removed	
<20	49 (39.5%)
≥20	72 (58.1%)
NA	3 (2.4%)

NA, not available.

We next calculated the lymph node metastasis rate according to pT distribution. As shown in **Table 3**, the lymph node metastasis rate was significantly higher when tumor lesions were more extensive, with a rate of 4.3%, 20.0%, and 58.8% for pT1, pT2, and pT3, respectively.

**Table 3:** Rates of lymph node metastasis according to pT status.

pT status	pN1	pN0	pNx	Rate of lymph node metastasis^1^
pT1 (n=93)	3	67	23	4.3%
pT2 (n=25)	4	16	5	20.0%
pT3 (n=52)	20	14	18	58.8%
Total (n=170)	27	97	46	27.8%

^1^Rate of lymph node metastasis = number of pN1/pN0+pN1.

pT, pathologic tumor status; pN, pathologic lymph node status; pN1, regional lymph node metastasis; pN0, no regional lymph node metastasis; pNx, lymph node metastasis not determined.

### Survival Analysis

#### In the Overall Cohort

**Figure 1:**
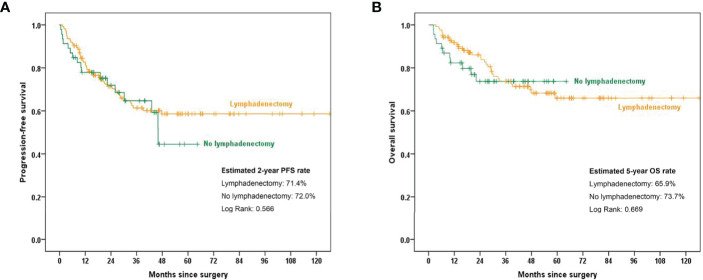
Kaplan–Meier curves showing 2-year PFS and 5-year OS rates between the lymphadenectomy group and no lymphadenectomy group. **(A)** PFS comparison in the overall cohort; **(B)** OS comparison in the overall cohort.PFS, progression-free survival; OS, overall survival.

The Kaplan–Meier curves shown in **Figure 1** indicate that, in the overall cohort, the estimated 2-year PFS was 71.4% and 72.0% in the lymphadenectomy group and no lymphadenectomy group, respectively (*p*=0.566). The estimated 5-year OS rates were 65.9% and 73.7% in the lymphadenectomy group and no lymphadenectomy group, respectively (*p*=0.669). No significant difference was found between the two groups.

#### Subgroup Analysis Stratified by FIGO Stage (Early and Advanced Stage)

**Figure 2:**
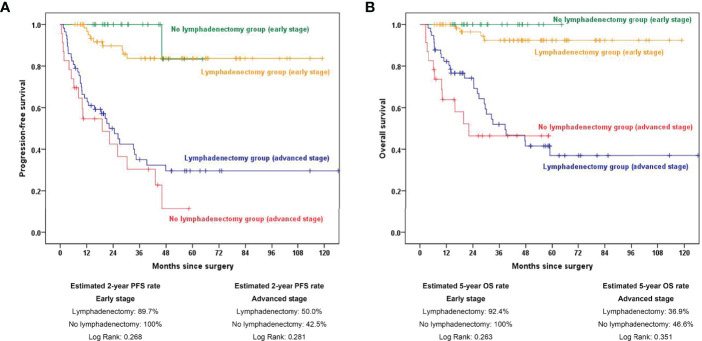
Kaplan–Meier curves showing 2-year PFS and 5-year OS rates stratified by early and advanced stage between the lymphadenectomy group and no lymphadenectomy group. **(A)** PFS comparison in subgroup analysis stratified by FIGO stage; **(B)** OS comparison in subgroup analysis stratified by FIGO stage. PFS, progression-free survival; OS, overall survival.

We next analyzed the role of retroperitoneal lymphadenectomy separately by stratifying all OCCC patients into early- and advanced-stage groups. The estimated 2-year PFS rates were 89.7% and 100.0% in the early-stage lymphadenectomy group and no lymphadenectomy group, respectively (*p*=0.256). The estimated 5-year OS rates were 92.4% and 100.0% in the early-stage lymphadenectomy group and no lymphadenectomy group, respectively (*p*=0.263). In advanced-stage patients, the estimated 2-year PFS rates were 50.0% in the lymphadenectomy group and 42.5% in the no lymphadenectomy group (*p*=0.281), and the estimated 5-year OS rates were 36.9% and 46.6% in the lymphadenectomy group and no lymphadenectomy group, respectively (*p*=0.351). The survival curves are displayed in **Figure 2**.

#### Subgroup Analysis in Patients With Clinically Negative Nodes

**Figure 3:**
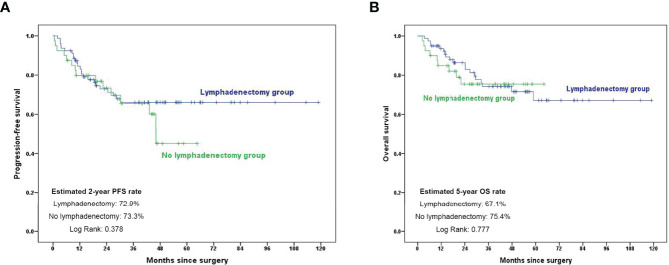
Kaplan–Meier curves showing 2-year PFS and 5-year OS rates in patients with clinically negative nodes. **(A)** PFS comparison in subgroup analysis in patients with clinically negative nodes; **(B)** OS comparison in subgroup analysis in patients with clinically negative nodes. PFS, progression-free survival; OS, overall survival.

Interestingly, we analyzed the role of retroperitoneal lymphadenectomy in patients with clinically negative nodes. As shown in **Figure 3**, there was no significant difference in the 2-year PFS and 5-year OS rate between the lymphadenectomy group and no lymphadenectomy group (*p* = 0.378 and 0.777, respectively).

### Univariate and Multivariate Analysis of the Predictors of Recurrence

As shown in **Table 4**, patients with advanced stage, VTE, Fagotti score ≥ 8, ascites, residual disease > 0, and less than four chemotherapy cycles had a shorter PFS by univariate analysis. Multivariate analysis showed that advanced stage (hazard ratio (HR), 3.082; 95% confidence interval (CI), 1.346–7.058), VTE (HR, 2.675; 95% CI, 1.112–6.433), ascites (HR, 2.354; 95% CI, 1.118–4.762), residual disease > 0 (HR, 8.128; 95% CI, 3.342-19.767), and less than four chemotherapy cycles (HR, 1.821; 95% CI, 1.015-3.268) were independent predictors of tumor recurrence, while retroperitoneal lymphadenectomy was not a significant factor influencing tumor recurrence.

**Table 4:** Univariate and multivariate analysis for progression-free survival in all OCCC patients.

Characteristics	N	Univariate		Multivariate	
		2-year PFS rate	*p* value	HR (95% CI)	*p* value
Age at diagnosis					
≤50	74	68.6%			
>50	96	73.7%	0.736		
FIGO stage					
IA-IIA	90	92.5%			
IIB-IVB	80	47.8%	<0.001	3.082 (1.346-7.058)	0.008
VTE					
No	155	73.0%			
Yes	15	54.5%	0.049	2.675 (1.112-6.433)	0.028
Fagotti score					
<8	152	77.9%			
≥8	18	0%	<0.001	1.764 (0.687-4.525)	0.238
Ascites					
None	102	87.1%			
Yes	63	49.4%	<0.001	2.354 (1.118-4.762)	0.014
Retroperitoneal lymphadenectomy					
No	46	72.0%			
Yes	124	71.4%	0.566	0.557 (0.265-1.168)	0.121
Residual disease					
NGR	145	81.8%			
RD >0	20	8.6%	<0.001	8.128 (3.342-19.767)	<0.001
Chemotherapy cycles					
≥4	127	88.7%			
<4	41	57.1%	0.011	1.821 (1.015-3.268)	0.044

OCCC, ovarian clear cell cancer; PFS, progression-free survival; HR, hazard ratio; CI, confidence interval; FIGO, International Federation of Gynecology and Obstetrics; VTE, venous thromboembolism; NGR, no gross residual disease; RD, residual disease.

## Discussion

Recent studies focusing on the role of retroperitoneal lymph node dissection have emerged following the results of the LION study (4). For advanced ovarian cancer patients, Fang et al. found that systematic lymphadenectomy did not improve survival in patients with no gross residual disease (NGR) or residual tumors measuring < 1 cm (5). Ting et al. showed that retroperitoneal lymph node dissection was not associated with a gain in overall- (OS) and progression-free survival (PFS) for patients with early-stage ovarian cancer (6). Chen et al. showed that retroperitoneal lymph node dissection was not significantly associated with improved prognosis for most stage I EOC patients, but may be necessary for the stage IC subtype (7). Bizzarri et al. showed that pelvic and para-aortic lymphadenectomy improved disease-free survival while having no impact on OS in apparent early-stage ovarian cancer patients (8). In our study, the results suggested that retroperitoneal lymphadenectomy provided no survival benefit in patients diagnosed with OCCC, no matter in the whole cohort or when subgroup analysis were performed stratified by early and advanced stage, or in patients with clinically negative nodes.

Although the results of an earlier study (9) showed that complete surgical staging involving pelvic and para-aortic lymphadenectomy appeared to improve survival in patients with stage I OCCC, more recent research showed no benefit (10). The recent studies including ours seem reasonable for the following reasons: First, for early-stage OCCC patients, the frequency of lymph node metastasis was much lower than other tumor subtypes according to previous studies. Heitz et al. studied the frequency of lymph node metastasis in patients with different tumor stages and histological subtypes who underwent pelvic and paraaortic lymphadenectomy. The results showed that 3.6% of OCCC patients with stage pT1a-pT2aM0 tumors had lymph node metastasis, while the rate was 71.6% in patients with high-grade serous ovarian cancer and 47.4% for high-grade endometrial cancer (11). Mahdi et al. estimated the prevalence of lymph node involvement in stage I OCCC patients from data from the SEER database, and the results showed that 61 (4.5%) of 1359 stage I OCCC patients were upstaged to FIGO stage III (12). In our study, the rate of lymph node metastasis was 4.3% in patients with stage pT1 disease (**Table 3**), similar to findings in these two previous studies. Second, regarding postoperative adjuvant chemotherapy, except for stage IA OCCC patients, for whom observation is feasible, both stage I and stage IIIA OCCC patients should receive postoperative chemotherapy, meaning that postoperative adjuvant therapy is almost unaffected by retroperitoneal lymphadenectomy. Furthermore, the rate of lymph node metastasis for all pT1 stage patients (< 5%) may also suggest a lower frequency in OCCC patients with stage pT1A tumors. Therefore, lymphadenectomy may accurately upstage only a small percentage (< 5%) of early-stage OCCC patients, indicating an extremely limited therapeutic role.

Some studies evaluating the number of resected lymph nodes in early OCCC, such as the study by Yuji et al. (13) showed that for patients with stage I OCCC, the group with ≥ 35 resected lymph nodes were correlated with better recurrence-free survival than those with < 35 resected lymph nodes. Harder et al. found a trend toward improved survival when more extensive lymphadenectomy (> 10 nodes) was performed, although there was no statistical significance (12). Matsuo et al. found that adequate lymphadenectomy was associated with a 15%–25% reduction in ovarian cancer mortality compared with inadequate lymphadenectomy (14). In our study, there was no survival difference between patients with < 20 vs ≥ 20 resected lymph nodes (**Supplementary Figure 1**).

A recent study of 410 advanced-stage ovarian cancer patients (including both serous and non-serous cancer) showed no significant difference in 5-year OS and 2-year PFS between the lymphadenectomy group and no lymphadenectomy group, while patients in the lymphadenectomy group had a higher incidence of infection (5). The study included patients with negative (n=288, 70.2%) and positive lymph nodes, and the results indicated no benefit with lymphadenectomy for both the entire cohort and when patients were stratified by lymph node clinical evaluation. ours is the first study investigating the therapeutic role of retroperitoneal lymphadenectomy in advanced-stage OCCC patients. As shown in **Table 3**, almost 60% of patients with stage pT3 OCCC had retroperitoneal lymph node metastasis, which was much higher than in patients with stage pT1 disease. Our results showed a negative prognostic role of lymphadenectomy in these patients.

In addition to advanced ovarian cancer patients undergoing primary debulking surgery, several recent studies have evaluated the role of lymphadenectomy in patients who underwent interval debulking surgery. A systematic literature review from Seidler et al, that included 1094 patients from six retrospective series, suggested no benefit of systematic lymphadenectomy during interval debulking surgery procedure on survival in node-negative, advanced-stage ovarian cancer patients (15). He et al. retrospectively analyzed the role of lymphadenectomy in advanced-stage ovarian cancer patients who underwent interval debulking surgery. Of the 303 patients included in the study, 163 (53.8%) patients achieved NGR, and 127 (41.9%) patients underwent lymphadenectomy. The results suggested no therapeutic value of lymphadenectomy, with both PFS and OS showing no statistical difference between the lymphadenectomy group and no lymphadenectomy group (16). In our study, we did not include patients received neoadjuvant chemotherapy for analysis to avoid bias.

Several limitations existed in our study. The first weakness was the low cases number. A more concrete analysis could be achieved with more cases enrolled, especially when subgroup analysis was performed in the study. Another limitation was that our study included patients with early-and advanced stage, optimal and sub-optimal surgery, and clinically positive and negative lymph nodes. The heterogeneity of the sample may also weaken the conclusion of our study. However, the results of the current study may provide evidence for designing a randomized clinical trial specifically for patients with ovarian clear cell cancer.

## Conclusions

In this retrospective study, we found no survival benefit of retroperitoneal lymphadenectomy in OCCC patients, both in the entire cohort and when subgroup analysis was performed. A prospective clinical trial is needed to confirm the present results.

## Data Availability Statement

The raw data supporting the conclusions of this article will be made available by the authors, without undue reservation.

## Ethics Statement

The studies involving human participants were reviewed and approved by Medical ethics committees of both Fudan University Zhongshan Hospital and Zhejiang Cancer Hospital. Written informed consent for participation was not required for this study in accordance with the national legislation and the institutional requirements.

## Author Contributions

SY, WG, and JZ contributed to conception and design of the study. PS, HS, and MX organized the database. SY and WG performed the statistical analysis. SY wrote the first draft of the manuscript. WG, PS, and WT wrote sections of the manuscript. All authors contributed to manuscript revision, read, and approved the submitted version.

## Funding

The study was funded by Zhongshan Development Program (XK-066).

## Conflict of Interest

The authors declare that the research was conducted in the absence of any commercial or financial relationships that could be construed as a potential conflict of interest.

## Publisher’s Note

All claims expressed in this article are solely those of the authors and do not necessarily represent those of their affiliated organizations, or those of the publisher, the editors and the reviewers. Any product that may be evaluated in this article, or claim that may be made by its manufacturer, is not guaranteed or endorsed by the publisher.
