# Supplementary material for: Therapeutic Role of Retroperitoneal Lymphadenectomy in 170 Patients With Ovarian Clear Cell Cancer
Source: Front Oncol. 2022 Jan 13;11:754149. doi: 10.3389/fonc.2021.754149 (PMC8791852; doi:10.3389/fonc.2021.754149)
Supplement: Supplementary file 1 [file DataSheet_1.pdf]

Supplementary Table 1. Clinical characteristics in 170 OCCC patients

Characteristics	n=170
Age at diagnosis	
≤ 50	74 (43.5%)
> 50	96 (56.5%)
Median preoperative CA125	140.7 U/ml
Median preoperative CA19-9	24.5 U/ml
FIGO Stage	
Stage I	89 (52.4%)
Stage II	20 (11.8%)
Stage III	49 (28.8%)
Stage IV	12 (7.1%)
Clinical lymph node status	
Positive	40 (23.5%)
Negative	119 (70.0%)
NA	11 (6.5%)
VTE	
Yes	15 (8.8%)
No	155 (91.2%)
Fagotti score	
0-4	130 (76.5%)
4-8	22 (12.9%)
≥ 8	18 (10.6%)
Ascites	
None	102 (60.0%)
Yes	63 (37.1%)
NA	5 (2.9%)
Residual disease	
NGR	145 (85.3%)
RD >0	20 (11.8%)
NA	5 (2.9%)
Chemotherapy	
Taxane + platinum	148 (87.1%)
Paclitaxel/Carboplatin	123
Docetaxel/Carboplatin	17
Paclitaxel/Cisplatin	8
Other platinum-based chemotherapy[a]	7 (4.1%)
Others[b]	2 (1.2%)
None	11 (6.5%)
NA	2 (1.2%)
Chemotherapy cycles	
0-3[c]	41 (24.1%)
≥ 4	127 (74.7)
NA	2 (1.2)

CA125, carbohydrate antigen 125; CA19-9, carbohydrate antigen 19-9; FIGO, International Federation of Gynecology and Obstetrics; VTE, venous thromboembolism; NGR, no gross residual disease; RD, residual disease; NA, not available.

[a] 7 patients received platinum-based chemotherapy without paclitaxel, including carboplatin single-agent chemotherapy (4), Pirarubicin/Cisplatin (1), Doxorubicin/Cisplatin (1) and Gemcitabine/cisplatin (1).

[b] 2 patients only received intraperitoneal chemotherapy.

[c] The reasons why these patients did not complete the standard treatment include drug intolerance, economic burden and low patient compliance.

Supplementary Figure 1. Kaplan–Meier curves showing estimates of 2-year PFS between patients with ≥20 and <20 resected lymph nodes.

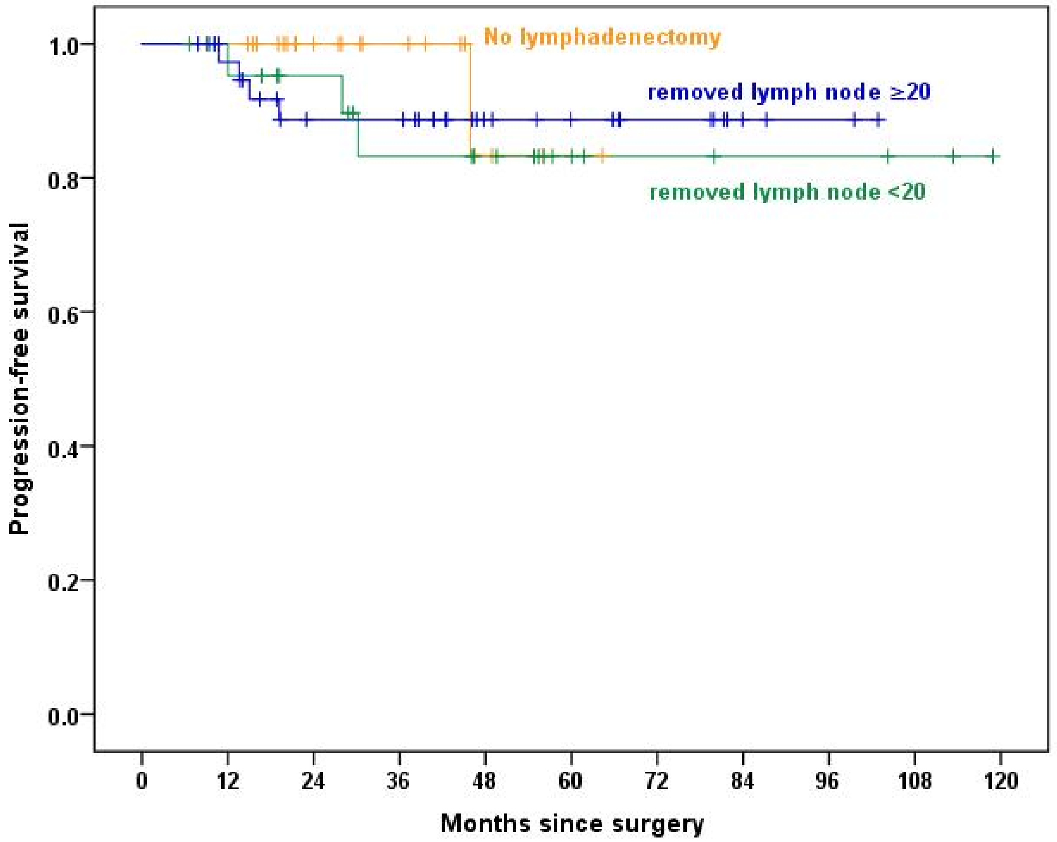

Estimated 2-year PFS rate

Removed lymph node <20: 95.2%

Removed lymph node ≥20: 88.7%

Log Rank: 0.735

PFS, progression-free survival
